# Assessment of new prognostic risk models for recurrence in patients with clinical stage I seminoma

**DOI:** 10.1111/bju.70041

**Published:** 2025-10-19

**Authors:** Angelina Strauch, Justine Schoch, Martin Hellmich, Hans Schmelz, Tim Nestler

**Affiliations:** ^1^ Department of Urology Federal Armed Forces Hospital Koblenz Koblenz Germany; ^2^ Institute of Medical Statistics and Computational Biology University Hospital of Cologne Cologne Germany; ^3^ Department of Urology University Hospital of Cologne Cologne Germany

**Keywords:** germ cell tumour, testicular cancer, seminoma, risk stratification, prognostic factors, adjuvant therapy, overtreatment

## Abstract

**Objectives:**

To evaluate two new prognostic risk models aimed at improving clinical decision‐making in patients with clinical stage (cS) I seminomatous testicular germ cell tumours (GCTs), 5%–30% of whom experience recurrence during surveillance after orchiectomy and may benefit from adjuvant therapy.

**Methods:**

In this exploratory study, patients with unilateral cS I seminoma, normalised serum tumour markers after orchiectomy, no adjuvant therapy and at least 12 months of follow‐up were included. Cox regression analysis was applied to evaluate the prognostic factors proposed by the Danish Testicular Cancer Database (DaTeCa) and the European Association of Urology (EAU), and recurrence probabilities were estimated using the Kaplan–Meier method.

**Results:**

Among 139 patients, 25 (18%) experienced recurrence within a median follow‐up of 37 months (95% confidence interval 47.9–63.1 months). Multivariable analysis confirmed only rete testis infiltration as an independent predictor of recurrence (*P* = 0.039), while other prognostic factors included in the DaTeCa risk model (i.e. hilar soft tissue invasion, rete testis infiltration, lymphovascular invasion, and elevated pre‐orchiectomy human chorionic gonadotropin and lactate dehydrogenase) and the EAU model (i.e. tumour size, rete testis infiltration and lymphovascular invasion) were not. However, 5‐year recurrence risks for the different risk groups, defined by the combination of prognostic factors, aligned well with the DaTeCa (no risk factors: 4% vs 6%; all risk factors: 67% vs 62%) and the EAU risk models (low [13% vs 8%] and intermediate risk [22% vs 20%]). A discrepancy was observed in EAU high‐risk cases (67% vs 44%), which was probably attributable to the very small number of patients in our high‐risk subgroup (*n* = 6 [4.3%]).

**Conclusion:**

The DaTeCa and EAU risk classification models demonstrated overall consistency in our exploratory cohort and may aid in identifying patients with cS I seminoma who are at high risk for recurrence and who might be candidates for adjuvant therapy. Further multicentre validation studies are needed.

**Abbreviations**
cS: clinical stage
DaTeCa: Danish Testicular Cancer database
EAU: European Association of Urology
GCT: germ cell tumour
LDH: lactate dehydrogenase
RFS: recurrence‐free survival

## Introduction

Testicular germ cell tumours (GCTs) are the most common malignancy in men aged 20–40 years [1, 2]. It has been reported that 79% of all GCT patients present with clinical stage (cS) I, showing no evidence of metastasis [3]. The most common histological subtype is pure seminoma, comprising 65% of cases [3, 4]. However, 5%–30% of patients with cS I seminoma harbour occult metastases and may develop recurrence [5, 6, 7].

Histological risk factors for recurrence include tumour size >4 cm and rete testis infiltration [8, 9]. Although these factors correlate with increased recurrence risk [8, 10], they fail to reliably distinguish between low‐ and high‐risk patients [10, 11, 12]. For tumours ≤4 cm, 5‐year recurrence‐free survival (RFS) rates range from 86.6% to 95.5%, compared to 73.0% to 82.6% for tumours >4 cm. Similarly, RFS rates range from 86.0% to 92.0% for patients without rete testis infiltration and from 74.9% to 79.5% with rete infiltration [10]. Given the low overall recurrence rate, the European Association of Urology (EAU) guidelines recommend surveillance as the preferred management strategy, minimising overtreatment and avoiding unnecessary chemo‐ or radiotherapy [9, 13].

To improve risk stratification, the EAU and Danish Testicular Cancer database (DaTeCa) introduced new prognostic models [14, 15]. The EAU defines tumour size (≥2 cm, >2–5 cm, >5 cm), rete testis infiltration, and lymphovascular invasion as prognostic factors for three new risk groups deemed to be at ‘low’, ‘intermediate’ and ‘high’ risk of recurrence [14]. The DaTeCa risk model incorporates hilar soft tissue invasion, rete testis infiltration, lymphovascular invasion, and elevated pre‐orchiectomy hCG and lactate dehydrogenase (LDH), assigning patients to six risk groups based on the number of risk factors present [15].

Both models aim to support more effective risk‐adapted management of cS I seminoma. This study evaluates these models in a contemporary cohort and investigates the following new prognostic markers: hCG and LDH density (preoperative tumour marker level relative to tumour size) and hCG and LDH ratio (preoperative to postoperative levels).

## Materials and Methods

### Study Population

This retrospective study included patients diagnosed with cS I seminoma between 2000 and 2023 at the Federal Armed Forces Hospital Koblenz, Germany. cS I was defined as presence of retroperitoneal lymph nodes with a short axial diameter <10 mm, as described in a previous study [16].

Staging involved contrast‐enhanced abdominal CT within 1 week after orchiectomy, with repeat imaging 6 weeks later for cases of questionable lymph node enlargement (cS IIA). Patients without suspicious lymph nodes on repeat imaging were classified as cS I.

Inclusion criteria were normalised serum tumour markers (hCG and LDH) after orchiectomy and no bilateral testicular tumours. Exclusion criteria were elevated α‐fetoprotein levels or any adjuvant therapy. A minimum follow‐up of 12 months was required. Patient selection details are shown in Fig. S1. We adhered to the Standards for Reporting of Diagnostic Accuracy Studies (STARD) recommendations [17] and the German testicular cancer S3 guideline [18], with follow‐up involving regular imaging and tumour marker assessments. Recurrence was defined as retroperitoneal lymph nodes whose short axial diameter had increased by ≥10 mm, presence of distant metastases, or elevated serum hCG. Isolated LDH increases without imaging evidence of metastasis were not considered recurrence.

Clinical data included patient age, follow‐up duration, pre‐/postoperative tumour markers (hCG and LDH) and recurrence rates. We also investigated two self‐defined prognostic factors: hCG and LDH density (ratio of preoperative tumour marker level to histological tumour size), inspired by PSA density in prostate cancer, and the hCG and LDH ratio (preoperative to postoperative tumour marker level). Pathological factors included tumour size (maximum dimension), rete testis infiltration and lymphovascular invasion. Hilar soft tissue invasion, a suggested prognostic factor in the DaTeCa model, was not further specified and was categorised under rete testis infiltration. Ethical approval was obtained (2021‐15756‐retrospective).

### Statistical Analyses

Statistical analyses were conducted using IBM SPSS Statistics System for Windows, v29.0 (Armonk, NY, USA). Categorical variables are presented as counts (*n*) and percentages, continuous variables as medians with interquartile ranges. Comparisons between patients with and without recurrence were conducted using the *t*‐test and Mann–Whitney *U*‐test.

Univariable and multivariable Cox regression analyses were performed to assess the prognostic factors proposed by the EAU and DaTeCa, with hazard ratios and 95% CI. Continuous variables included pre‐orchiectomy hCG, LDH, and tumour size, while rete testis infiltration and lymphovascular invasion were dichotomised. *P* values < 0.05 were taken to indicate statistical significance.

Kaplan–Meier curves illustrated RFS, censoring patients at their last follow‐up. Risk groups were categorised according to the EAU and DaTeCa criteria, with cumulative recurrence probabilities at 1 and 5 years calculated with 95% CI. These time periods were chosen to facilitate comparability with the original studies.

## Results

### Patient Cohort

A total of 139 patients with cS I seminoma were included in the study (Fig. S1). The clinical and pathological characteristics of the cohort are summarised in Table 1. Within a median follow‐up of 37 months, 25 patients (18%) experienced recurrence. The prognostic risk factors defined by the EAU – rete testis infiltration, tumour size (>4 cm) and lymphovascular invasion – were present in 30%, 26% and 22% of patients, respectively. DaTeCa risk factors – rete testis infiltration, lymphovascular invasion, and elevated preoperative hCG and LDH – were present in 30%, 22%, 30% and 21% of patients, respectively. Significant differences were observed between patients with and without recurrence only concerning testicular tumour size (*P* = 0.002) and rete testis infiltration (*P* = 0.009), both being more prevalent in those with recurrence (Table 2). Most patients with recurrence presented with retroperitoneal lymph node metastasis (23 [92%]), while only two patients (8%) had distant metastasis with mediastinal and pulmonal tumours.

**Table 1:** Clinical and histopathological characteristics of the study cohort (*n* = 139).

Variable	
Number of patients, *n* (%)	139 (100)
**Patient age at diagnosis**	
Median (IQR) years	36 (30, 44)
**Testicular tumour size**	
Median (IQR) mm	25 (18, 40)
Tumour size >40 mm, *n* (%)	36 (26)
Tumour size ≤40 mm, *n* (%)	103 (74)
**Lymphovascular invasion, *n* (%)**	
Present	31 (22)
Absent	108 (78)
**Infiltration of rete testis, *n* (%)**	
Present	42 (30)
Absent	97 (70)
**hCG (normal <2 mIU/mL)**	
Normal, *n* (%)	98 (70)
Elevated, *n* (%)	41 (30)
Median (IQR) hCG in elevated cases, mIU/mL	3.2 (2.5, 5.4)
**hCG ratio**	
Median (IQR)	2.23 (0.95, 5.50)
Unknown	52 (37)
**Median (IQR) hCG density**	0.04 (0.02, 0.09)
**LDH (normal < 225 U/L)**	
Normal, *n* (%)	110 (79)
Elevated, *n* (%)	29 (21)
Median (IQR) LDH in elevated cases, U/L	266 (246, 292)
**LDH ratio**	
Median (IQR)	1.11 (1.01, 1.25)
Unknown	44 (32)
**Median (IQR) LDH density**	7.40 (4.95, 10.28)
**Follow‐up**	
Median (IQR) months	37 (25, 74)
**Recurrence, *n* (%)**	25 (18)
**Deaths, *n***	0

IQR, interquartile range; LDH, lactate dehydrogenase.

**Table 2:** Clinical and histopathological characteristics of the study cohort (*n* = 139) for patients with and without recurrence.

Variable	Patients without recurrence	Patients with recurrene	*P*
Number of patients, *n* (%)	114 (82)	25 (18)	
**Patient age at diagnosis**			
Median (IQR) years	36 (30, 45)	41 (29, 45)	0.928
**Testicular tumour size**			0.002
Median (IQR) mm	25 (18, 37)	32 (24, 58)	0.077
Tumour size >40 mm, *n* (%)	26 (23)	10 (40)	
Tumour size <40 mm, *n* (%)	88 (77)	15 (60)	
**Lymphovascular invasion, *n* (%)**			
Present	23 (20)	8 (32)	0.200
Absent	91 (80)	17 (68)	
**Infiltration of rete testis, *n* (%)**			
Present	29 (25)	13 (52)	0.009
Absent	85 (75)	12 (48)	
**hCG (normal <2 mIU/mL)**			
Normal, *n* (%)	83 (73)	15 (60)	0.389
Elevated, *n* (%)	31 (27)	10 (40)	
Median (IQR) in elevated cases, mIU/mL	3.1 (2.5, 4.1)	4.60 (2.48, 9.21)	
**hCG ratio**			
Median (IQR)	2.02 (0.94, 5.45)	2.37 (0.96, 6.45)	0.960
Unknown, *n* (%)	45 (39)	7 (25)	
**hCG density**			
Median (IQR)	0.05 (0.02, 0.09)	0.03 (0.03, 0.10)	0.942
**LDH (normal < 225 U/L)**			
Normal, *n* (%)	94 (82)	16 (64)	0.056
Elevated, *n* (%)	20 (18)	9 (36)	
Median (IQR) in elevated cases, U/L	272 (248, 292)	250 (233, 293)	
**LDH ratio**			
Median (IQR)	1.11 (1.02, 1.24)	1.12 (0.97, 1.38)	0.322
Unknown, *n* (%)	38 (33)	6 (24)	
**LDH density**			
Median (IQR)	7.45 (5.20, 10.50)	6.91 (3.77, 9.76)	0.085
**Follow‐up**			
Median (IQR) months	41 (27, 78)	24 (17, 57)	

*P* values were calculated using the Mann–Whitney *U‐* and *t*‐test. IQR, interquartile range, LDH, lactate dehydrogenase.

### Established Risk Factors

The 5‐year RFS rate for patients with tumours ≤4 cm was 85%, for tumours >4 cm we calculated a rate of 72% (Fig. S2A). Patients without rete testis infiltration had a 5‐year RFS of 88%, whereas the 5‐year RFS for patients with rete testis infiltration was 69% (Fig. S2B).

### New Prognostic Factors

Univariable Cox regression analysis for RFS was conducted to assess the prognostic factors proposed by the EAU and DaTeCa (Table S1). Our results indicated a significant association with recurrence only for tumour size as a continuous variable (*P* = 0.004) and rete testis infiltration (*P* = 0.006). Multivariable Cox regression analysis (Table S2), including all prognostic factors from the univariable analysis, showed that rete testis infiltration was the only independent predictor of recurrence (*P* = 0.039).

### Risk Groups

According to the EAU publication, 87 patients (63%) in our study cohort were in the low‐risk group, 46 (33%) were in the intermediate‐risk group, and six (4%) were in the high‐risk group (Table S3). Based on the DaTeCa classification, 49 patients (35%) were in the lowest‐risk group with no risk factors. Patients with one, two, three and four risk factors accounted for 56 (40%), 18 (13%), 13 (9%) and three patients (2%), respectively. No patients were categorised in the highest DaTeCa risk group, as hilar soft tissue invasion was not considered a separate risk factor (Table S3).

**Fig. 1:**
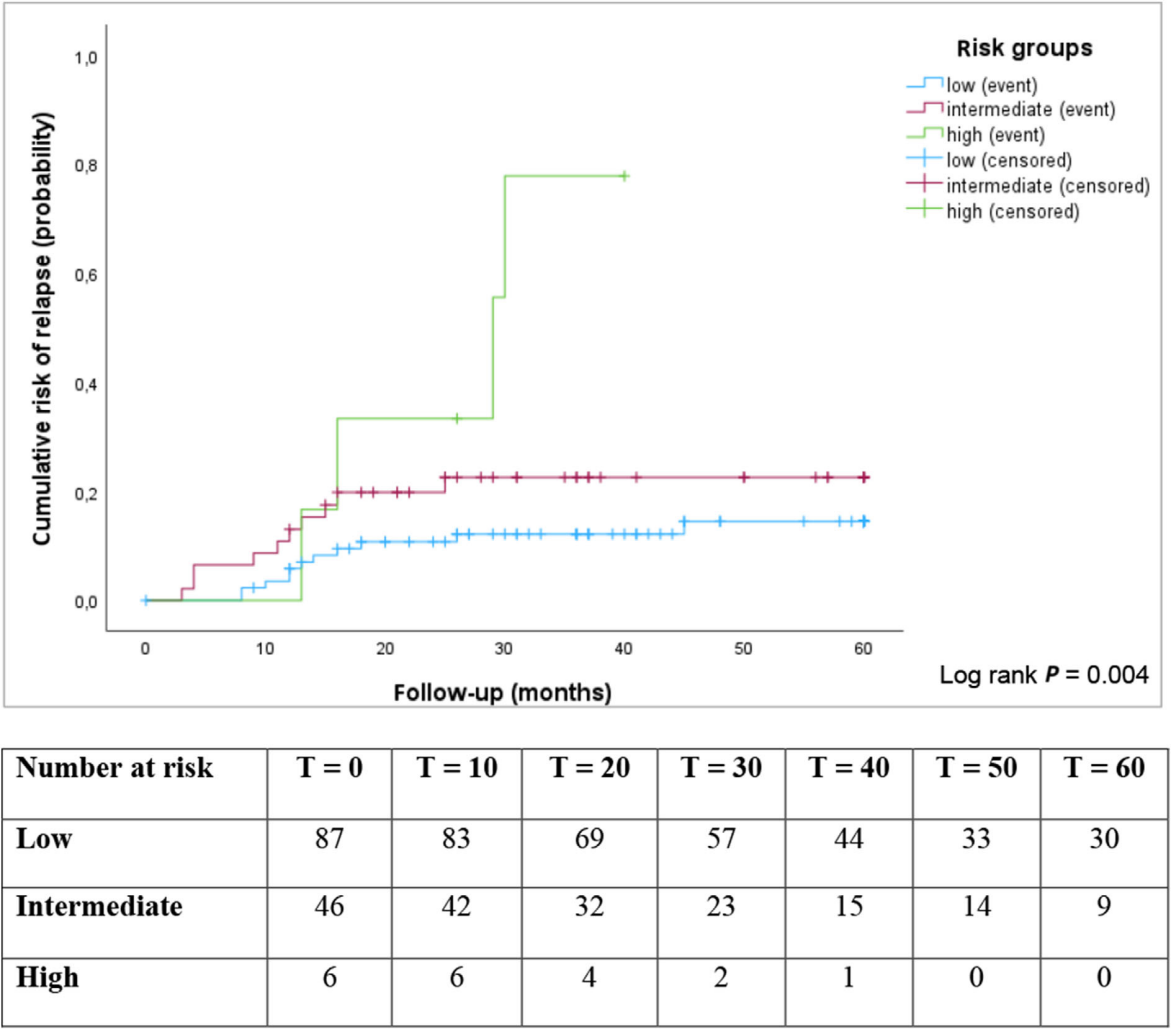
Kaplan–Meier curve representing cumulative risk of recurrence of the study cohort in the first 5 years after diagnosis, according to European Association of Urology risk group. Event = recurrence; censored = last follow‐up without recurrence.

**Fig. 2:**
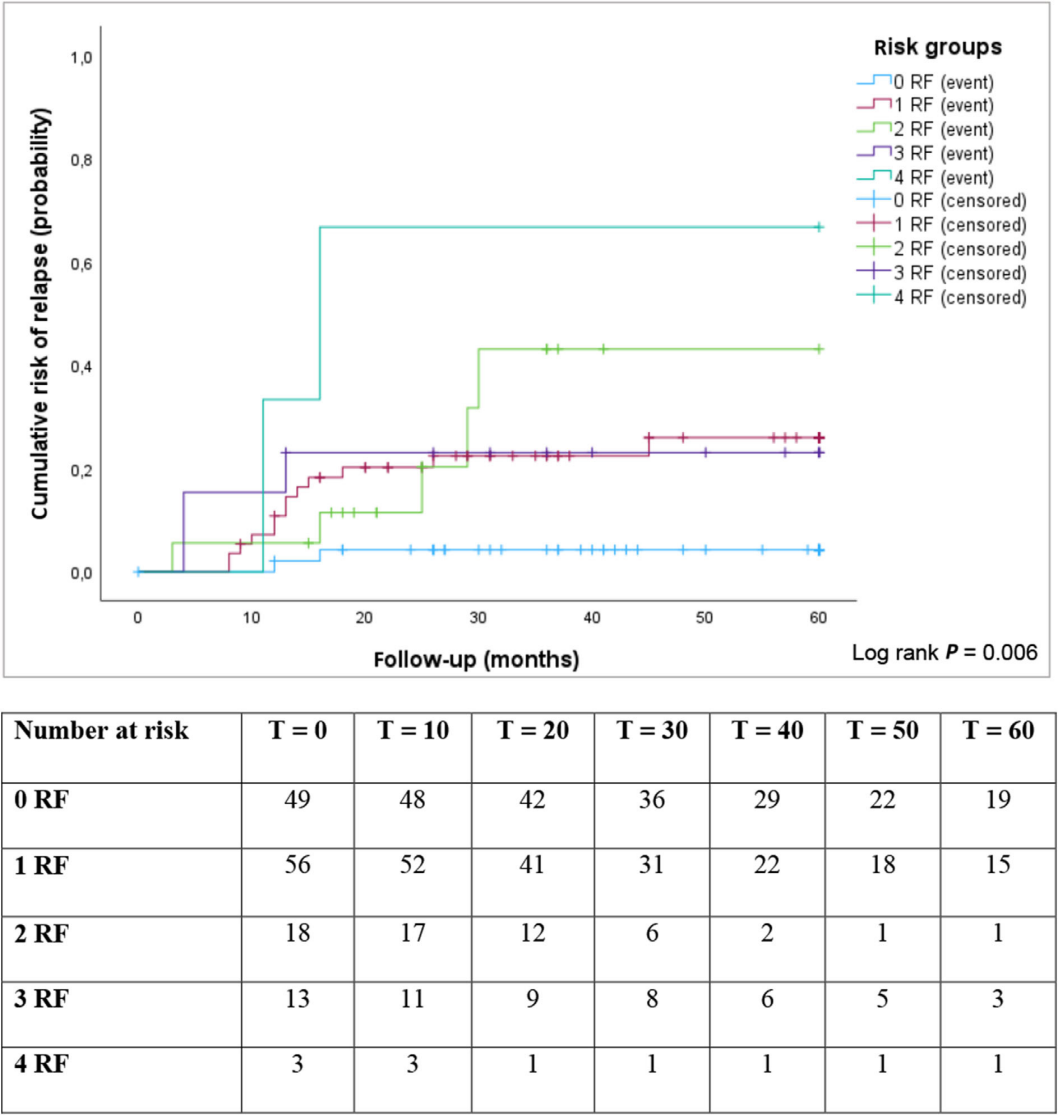
Kaplan–Meier curve representing cumulative risk of recurrence of the study cohort in the first 5 years after diagnosis, according to Danish Testicular Cancer database (DaTeCa) risk group. Event = recurrence; censored = last follow‐up without recurrence. RF, risk factor.

The calculated cumulative probabilities of recurrence at 1 and 5 years after orchiectomy for each risk group are also presented in Table S3. According to the EAU classification, the 5‐year recurrence risk increased from 13% in the low‐risk group to 67% in the high‐risk group. Using the DaTeCa model, the 5‐year recurrence risk rose from 4% in patients with no risk factors to 67% in those with four risk factors, while those with one to three risk factors had a similar 5‐year recurrence risk of 23%–28%. This means that four out of six patients in the high‐risk group according to the EAU model and two out of three patients in the high‐risk group according to the DaTeCa model experienced a recurrence. Kaplan–Meier survival curves for the EAU and DaTeCa risk groups (Figs 1 and 2) indicate that most recurrences occurred within the first 2 years, with 20 patients (80%) experiencing recurrence during this period. There was no late recurrence after 5 years.

### ß‐hCG and LDH Density and Ratio

The additional potential risk factors, hCG and LDH density and ratio, showed no significant differences between patients with and without recurrence (Table 2). Consequently, neither univariate nor multivariable Cox regression analysis indicated these variables as independent predictors of recurrence (Tables S1 and S2).

## Discussion

In this study, we aimed to evaluate the new risk models from the DaTeCa and EAU for recurrence in patients with cS I seminoma, using a small, but independent and contemporary cohort. The distribution of the EAU prognostic factors (rete testis infiltration, tumour size >4 cm and lymphovascular invasion) in our cohort (30%, 26% and 22%, respectively) was similar to the original study (37%, 30% and 12%), with lymphovascular invasion being more prevalent in our cohort. Among the DaTeCa risk factors (rete testis infiltration, lymphovascular invasion, elevated preoperative hCG and LDH [without hilar soft tissue invasion]), lymphovascular invasion was more frequent in our cohort, whereas rete testis infiltration and elevated LDH were less common; in our cohort the distribution was 30%, 22%, 30% and 21%, respectively, compared to 46%, 13%, 29% and 38% in the original study.

### Established Risk Factors

In our study cohort, only tumour size >4 cm and rete testis infiltration significantly differed between patients with and without recurrence. In multivariable Cox regression analysis, rete testis infiltration remained the sole independent predictor of recurrence, consistent with its inclusion in both the EAU and the DaTeCa models. The 5‐year RFS aligned with literature expectations: 85% (86.6%–95.5%) for tumours ≤4 cm and 72% (73.0%–82.6%) for tumours >4 cm, and 88% (86.0%–92.0%) for absence vs 69% (74.9%–79.5%) for presence of rete testis infiltration [10]. Although the median follow‐up in our study was 37 months, 5‐year recurrence rates were applied to enable a better comparison with the original EAU and DaTeCa studies. Notably, only one of 25 patients (4%) experienced recurrence beyond 37 months.

The risk factors tumour size and rete testis infiltration remain under discussion due to insufficient and inconsistent literature. For tumour size, challenges include the lack of standardised histopathological assessment and difficulties associated with multifocal tumours. In clinical practice, tumour size >4 cm is treated as a risk factor for seminomas at cS I, based on data from Warde et al. [19], who identified this as the median tumour size in their cohort. Since then, various studies have applied different cut‐off values [10, 12] or identified tumour size as a continuous variable associated with recurrence risk [20]. In our study, tumour size may not have been identified as a significant prognostic factor because patients who received adjuvant therapy after orchiectomy – typically those with at least one established risk factor for seminoma (rete testis infiltration or tumour size >4 cm) – were not included.

Regarding rete testis infiltration, while our study, like some other studies, confirmed it as an independent predictor of recurrence [10, 11, 12], the lack of clear definitions and standardisation in pathological evaluation complicates comparability among studies [10].

### New Prognostic Factors and Risk Groups

The additional prognostic factors for recurrence from the EAU and DaTeCa (lymphovascular invasion, elevated hCG and LDH) did not demonstrate significant predictive value in our cohort, aligning with prior studies [8, 11, 12, 19]. hCG and LDH density and ratio, which were self‐defined and have not yet been investigated in predicting occult metastasis in seminoma, also showed no significant differences between recurrence‐free patients and those with recurrence. However, risk group classifications showed consistency with our cohort for 5‐year recurrence risks: DaTeCa (no risk factors: 6% vs 4%; all risk factors: 62% vs 67%) and EAU (low risk: 8% vs 13%; intermediate risk: 20% vs 22%). High‐risk patients showed a higher recurrence risk in our cohort compared to EAU with 67% vs 44%, possibly due to the very small size of this group in our cohort with 4.3% of patients, which is also a limitation of this study.

### Implications for Clinical Practice

The EAU guideline and the German S3 guideline for testicular cancer recommend surveillance for patients with cS I seminoma regardless of risk factors [9, 18]. This approach is based on the observation that recurrence rates in patients with seminoma are generally low and patients under surveillance who experience recurrence achieve the same survival rates with appropriate delayed treatment as those who receive initial therapy [21]. Additionally, because of the young age of the patients and the high cure rates, the focus is increasingly on avoiding unnecessary therapy‐related long‐term side effects [13, 22].

Given the challenges associated with the established histological risk factors and the fact that only 22%–28% of patients in the low‐ and intermediate‐risk groups (EAU) or in the 0–3 risk factor groups (DaTeCa) experience recurrence within 5 years (similar to the original studies), surveillance should remain the preferred approach for these groups, which include the majority of patients (>95%). However, the new risk models could be particularly useful in guiding decisions regarding adjuvant therapy in the small high‐risk group. The objective is not to eliminate all recurrences but rather to achieve fewer recurrences while avoiding unnecessary treatments. Thus, a classification into low‐risk and high‐risk patients might be sufficient. From our perspective, the EAU classification, which includes fewer risk groups and relies exclusively on already established risk factors, is preferable to the DaTeCa classification. Non‐established risk factors, such as hilar soft tissue invasion, would be more challenging to implement in clinical practice.

Notably, in the high‐risk group as defined by the EAU, only 44% of patients in the original study experienced recurrence, compared to 67% in our analysis. This discrepancy warrants further investigation in multicentre studies, particularly due to the limited number of high‐risk patients in current research. In the original EAU study, only 2.3% of patients (*n* = 23) were in this group, compared to 4.3% (*n* = 6) in our cohort. Similarly, in the highest risk group defined by the DaTeCa, 2% of patients (*n* = 15) were included, while in our cohort, this was 2.2% (*n* = 3). Otherwise, even with the new risk models, a substantial proportion of patients, ranging from one‐third to over half, might still undergo overtreatment. To address this, the high‐risk group would need to be further refined, for example, with higher threshold values determining the risk factors.

Additional strategies are already emerging for individualised decision‐making, particularly to avoid overtreatment. Current GCT guidelines already incorporate re‐staging marker‐negative non‐seminoma patients after 6 weeks to distinguish true lymph node metastases from reactive postoperative retroperitoneal lymph node enlargement [9, 18]. Furthermore, primary retroperitoneal lymphadenectomy is being investigated as an alternative to radiotherapy or chemotherapy for patients with seminoma in cS IIA/B [23, 24]. Also, approaches combining de‐escalated chemotherapy and radiotherapy in patients with cS IIA/B seminoma have been analysed [25].

Standardisation and comparability of histological evaluations, especially for tumour size and rete testis infiltration, could benefit from artificial intelligence techniques such as deep learning [26]. Additionally, novel approaches such as using microRNA as a serum biomarker show promise. MicroRNA has a higher sensitivity and specificity than established histological risk factors and can predict occult metastasis in patients with seminoma independently of CT imaging during follow‐up [27, 28, 29].

The study was limited by its retrospective design and the lack of centralised pathology review, as tissue samples from patients diagnosed over 10 years ago were no longer available. Consequently, hilar soft tissue invasion, which was reported in 19% of patients in the original DaTeCa study, could not be evaluated as a separate risk factor. However, hilar soft tissue invasion is not a routinely assessed factor in clinical practice and is not required by current guidelines. Additionally, the number of high‐risk patients was limited, because 31 of 192 patients (Fig. S1) who received adjuvant therapy, which was applied when at least one established risk factor for seminoma was present (rete testis infiltration or tumour size >4 cm), were excluded.

## Conclusion

The DaTeCa and EAU risk classification models demonstrated overall consistency in our small cohort and may aid in identifying cS I seminoma patients at high recurrence risk who could benefit from adjuvant therapy. However, since the majority of patients have a low or intermediate recurrence risk, surveillance should remain the preferred approach for most cS I seminoma cases. Given that most proposed prognostic factors were not confirmed in our multivariable analysis and that the number of high‐risk patients was limited, both in our cohort and in the original studies, further validation in multicentre studies is warranted.

## Author Contributions

Conceptualisation: Angelina Strauch and Tim Nestler. Data curation: Angelina Strauch and Tim Nestler. Formal analysis: Angelina Strauch, Martin Hellmich and Tim Nestler. Supervision: Tim Nestler. Writing – original draft, Angelina Strauch and Tim Nestler. Writing – review and editing: Justine Schoch, Martin Hellmich and Hans Schmelz.

## Funding

This research did not receive any specific grant from funding agencies in the public, commercial, or not‐for‐profit sectors.

## Disclosure of Interests

The authors have no relevant financial or nonfinancial interests to disclose.

## Ethics Statement

The study complies with the Declaration of Helsinki, and local ethics committee approval was obtained (2021‐15 756‐retrospektiv). All authors adhere to the Ethical Policies of the journal. The present study was retrospective; for this type of study formal consent is not required.

## Supporting information


**Fig. S1.** Patient accrual according to Standards for Reporting of Diagnostic Accuracy Studies (STARD) recommendations (17).
**Fig. S2.** (A) Kaplan–Meier curve representing cumulative risk of recurrence of the study cohort in the first 5 years after diagnosis, depending on the risk factor tumour size. (B) Kaplan–Meier curve representing cumulative risk of recurrence of the study cohort in the first 5 years after diagnosis, depending on the risk factor rete testis infiltration.
**Table S1.** Univariable cox regression analysis for time to recurrence.
**Table S2.** Multivariable cox regression analysis for time to recurrence.
**Table S3.** Cumulative probability of recurrence of the study cohort at 1‐ and 5‐years after orchiectomy for EAU and DaTeCa risk groups, respectively.

## Data Availability

The data of this study are available from the corresponding author upon reasonable request.
