# Supplementary material for: Assessment of new prognostic risk models for recurrence in patients with clinical stage I seminoma
Source: BJU Int. 2025 Oct 19;137(1):138–45. doi: 10.1111/bju.70041 (PMC12690351; doi:10.1111/bju.70041)
Supplement: Supplementary file 1 — Fig. S1. Patient accrual according to Standards for Reporting of Diagnostic Accuracy Studies (STARD) recommendations (17). Fig. S2. (A) Kaplan–Meier curve representing cumulative risk of recurrence of the study cohort in the first 5 years after diagnosis, depending on the risk factor tumour size. (B) Kaplan–Meier curve representing cumulative risk of recurrence of the study cohort in the first 5 years after diagnosis, depending on the risk factor rete testis infiltration. Table S1. Univariable cox regression analysis for time to recurrence. Table S2. Multivariable cox regression analysis for time to recurrence. Table S3. Cumulative probability of recurrence of the study cohort at 1‐ and 5‐years after orchiectomy for EAU and DaTeCa risk groups, respectively. [file BJU-137-138-s001.docx]

**Table S1:** Univariable cox regression analysis for time to relapse.

| **Variable** | **HR (95% CI)** | ***p*** |
| --- | --- | --- |
| **Patient age** | 1.003 (0.968 – 1.038) | 0.887 |
| **Tumor size** | 1.030 (1.010 – 1.050) | **0.004** |
| **Lymphovascular invasion** | 1.661 (0.716 – 3.853) | 0.237 |
| **Rete testis infiltration** | 3.004 (1.366 – 6.608) | **0.006** |
| **hCG** | 1.026 (0.963 – 1.094) | 0.430 |
| **hCG ratio** | 0.997 (0.943 – 1.053) | 0.906 |
| **hCG density** | 1.152 (0.062 – 21.251) | 0.924 |
| **LDH** | 1.006 (0.999 – 1.012) | 0.075 |
| **LDH ratio** | 3.099 (0.379 – 25.307) | 0.201 |
| **LDH density** | 0.910 (0.812 – 1.021) | 0.109 |

*hCG = human chorionic gonadotropin, HR = hazard ratio, CI = confidence interval, IQR = interquartile range, LDH = lactate dehydrogenase*

**Table S2:** Multivariable cox regression analysis for time to relapse.

| **Variable** | **HR (95% CI)** | ***p*** |
| --- | --- | --- |
| **Patient age** | 1.002 (0.966 – 1.039) | 0.930 |
| **Tumor size** | 1.011 (0.974 – 1.050) | 0.555 |
| **Lymphovascular invasion** | 0.704 (0.262 – 1.895) | 0.487 |
| **Rete testis infiltration** | 2.675 (1.048 – 6.824) | **0.039** |
| **hCG** | 1.168 (0.446 – 3.061) | 0.752 |
| **hCG ratio** | 0.976 (0.915 – 1.041) | 0.465 |
| **hCG density** | 1.209 (0.057 – 25.533) | 0.903 |
| **LDH** | 2.282 (0.839 – 6.206) | 0.106 |
| **LDH ratio** | 5.875 (0.139 – 247.604) | 0.354 |
| **LDH density** | 0.944 (0.790 – 1.128) | 0.525 |

*hCG = human chorionic gonadotropin, HR = hazard ratio, CI = confidence interval, IQR = interquartile range, LDH = lactate dehydrogenase*

**Table S3:** Cumulative probability of relapse of the study cohort at 1- and 5-years after orchiectomy for EAU and DaTeCa risk groups, respectively.

| **Risk groups** | **Cumulative probability of relapse and 95% CI** | |
| --- | --- | --- |
|  | | |
| **EAU (139 patients)** | **at 1 year** | **at 5 years** |
| Low risk  87 patients (62.6%) | 0.06 (0.02 – 0.13) | 0.13 (0.07 – 0.22) |
| Intermediate risk  46 patients (33.1%) | 0.13 (0.05 – 0.26) | 0.22 (0.11 – 0.36) |
| High risk  6 patients (4.3%) | 0 (0 – 0.46) | 0.67 (0.22 – 0.96) |
|  | | |
|  | | |
| **DaTeCa (139 patients)** | **at 1 year** | **at 5 years** |
| No risk factors  49 patients (35.2%) | 0.02 (0.001 – 0.11) | 0.04 (0.005 – 0.14) |
| 1 risk factor  56 patients (40.3%) | 0.11 (0.04 – 0.22) | 0.23 (0.13 – 0.36) |
| 2 risk factors  18 patients (12.9%) | 0.06 (0.001 – 0.27) | 0.28 (0.10 – 0.54) |
| 3 risk factors  13 patients (9.4%) | 0.15 (0.02 – 0.45) | 0.23 (0.05 – 0.54) |
| 4 risk factors  3 patients (2.2%) | 0.33 (0.008 – 0.91) | 0.67 (0.10 – 0.99) |
| 5 risk factors  0 patients | NA | NA |

*CI = confidence interval, DaTeCa = Danish Testicular Cancer database, EAU = European Association of Urology, NA = not available*

cSI seminoma patients at our institution from 2000 to 2023 (n = 192)

Patients with adjuvant therapy after ablatio testis (n = 31)

Possible patient cohort (n = 161)

**Included patients (n = 139)**

No follow-up ≥12 months available

(n = 22)

**Figure S1:** Patient accrual according to Standards for Reporting of Diagnostic Accuracy Studies (STARD) recommendations (17).

*cS = clinical stage*


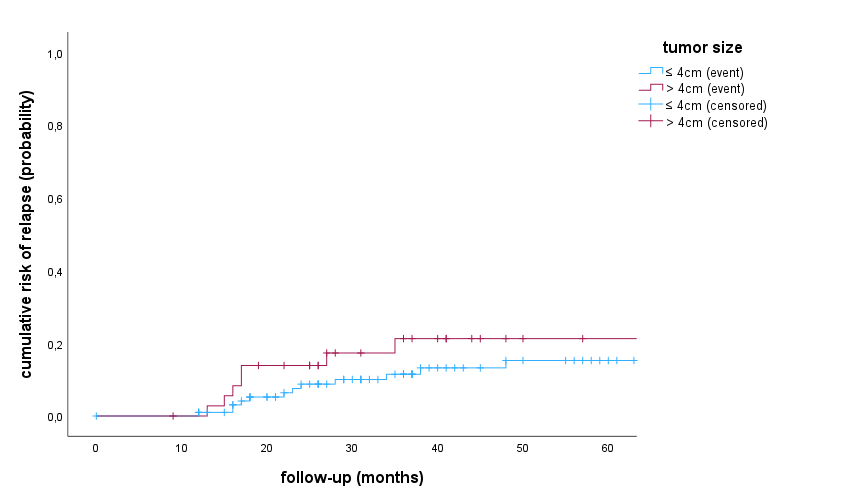


Log rank *p* = 0.062

**Figure S2A:** Kaplan-Meier curve representing cumulative risk of relapse of the study cohort in the first 5 years after diagnosis, depending on the risk factor tumor size.

*
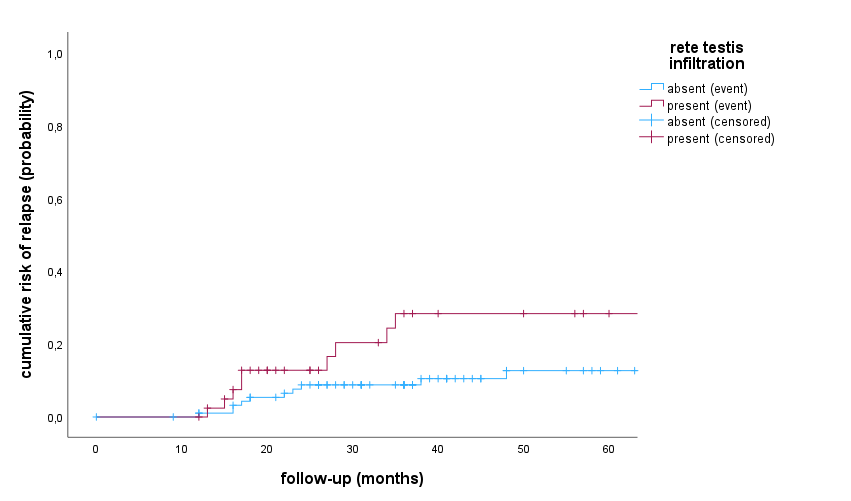
*

Log rank *p* = 0.004

**Figure S2B:** Kaplan-Meier curve representing cumulative risk of relapse of the study cohort in the first 5 years after diagnosis, depending on the risk factor rete testis infiltration.
